# Metabolic Engineering of *Corynebacterium glutamicum* for Sustainable Production of the Aromatic Dicarboxylic Acid Dipicolinic Acid

**DOI:** 10.3390/microorganisms10040730

**Published:** 2022-03-29

**Authors:** Lynn S. Schwardmann, Aron K. Dransfeld, Thomas Schäffer, Volker F. Wendisch

**Affiliations:** 1 Genetics of Prokaryotes, Faculty of Biology and CeBiTec, Bielefeld University, Universitätsstr. 25, 33615 Bielefeld, Germany; l.schwardmann@uni-bielefeld.de (L.S.S.); aron.dransfeld@uni-bielefeld.de (A.K.D.); 2 Multiscale Bioengineering, Technical Faculty and CeBiTec, Bielefeld University, Universitätsstr. 25, 33615 Bielefeld, Germany; thomas.schaeffer@uni-bielefeld.de

**Keywords:** dipicolinic acid, *Corynebacterium glutamicum*, metabolic engineering, sustainable production, nonsterile fermentation, phosphite

## Abstract

Dipicolinic acid (DPA) is an aromatic dicarboxylic acid that mediates heat-stability and is easily biodegradable and non-toxic. Currently, the production of DPA is fossil-based, but bioproduction of DPA may help to replace fossil-based plastics as it can be used for the production of polyesters or polyamides. Moreover, it serves as a stabilizer for peroxides or organic materials. The antioxidative, antimicrobial and antifungal effects of DPA make it interesting for pharmaceutical applications. In nature, DPA is essential for sporulation of *Bacillus* and *Clostridium* species, and its biosynthesis shares the first three reactions with the L-lysine pathway. *Corynebacterium glutamicum* is a major host for the fermentative production of amino acids, including the million-ton per year production of L-lysine. This study revealed that DPA reduced the growth rate of *C. glutamicum* to half-maximal at about 1.6 g·L^−1^. The first de novo production of DPA by *C. glutamicum* was established by overexpression of dipicolinate synthase genes from *Paenibacillus sonchi* genomovar *riograndensis* SBR5 in a *C. glutamicum* L-lysine producer strain. Upon systems metabolic engineering, DPA production to 2.5 g·L^−1^ in shake-flask and 1.5 g·L^−1^ in fed-batch bioreactor cultivations was shown. Moreover, DPA production from the alternative carbon substrates arabinose, xylose, glycerol, and starch was established. Finally, expression of the codon-harmonized phosphite dehydrogenase gene from *P. stutzeri* enabled phosphite-dependent non-sterile DPA production.

## 1. Introduction

DPA or pyridine-2,6-dicarboxylic acid is a naturally occurring aromatic dicarboxylic acid. Due to its metal-chelating properties, it serves as ligand for lanthanides [1], e.g., to transfer enhanced stability to micelles [2], and is used in complexes with transition metals, such as copper [3], or the actinide uranium. The latter provides the basis for potential applications in nuclear energy systems [4]. DPA also prevents calcium salt precipitation in silver halide photographic solutions [5]. In chemical industry, it serves as precursor for the synthesis of pyridines and piperidines [6] and as a stabilizer of peroxides in aqueous solutions, e.g., in peroxycarboxylic acids and gels of organic peroxides by reducing the decomposition rate [7,8,9]. Furthermore, its antioxidative effect makes DPA interesting for use in pharmacy [10,11]. It confers antimicrobial activity, enabling its application as an antimicrobial activity reagent [12,13], and the recently observed antifungal activity against canker pathogens reduced the symptoms of *Valsa pyri* infections of pear trees, showing its potential for the management of valsa canker [14]. DPA is a non-toxic dicarboxylic acid that shares some properties, including easy biodegradability and heat-stability, with diamines, with which it can be copolymerized to polyamides [15,16,17]. DPA has the potential to contribute to a more sustainable production of bio-based polymers by replacing fossil-based monomers as starting material as shown for pyridine based polyesters [10,16,18], furandicarboxylic acid based polyesters [19] and polyamides [20,21,22].

Naturally, DPA occurs as a secondary metabolite in endospores of gram-positive bacteria, mainly aerobic *Bacillus* and anaerobic *Clostridium* species. It is essential for initiation of sporulation under environmentally stressful conditions, such as nutrient starvation, heat, radiation or the presence of toxic compounds and stability of the endospore [23]. In the endospore, it occurs in a chelate with calcium ions and accounts for approximately 10% of the endospore dry weight in *Bacillus subtilis* [24]. Thus, DPA mediates heat resistance [25] and prevents DNA denaturation of the endospore [26].

**Figure 1:**
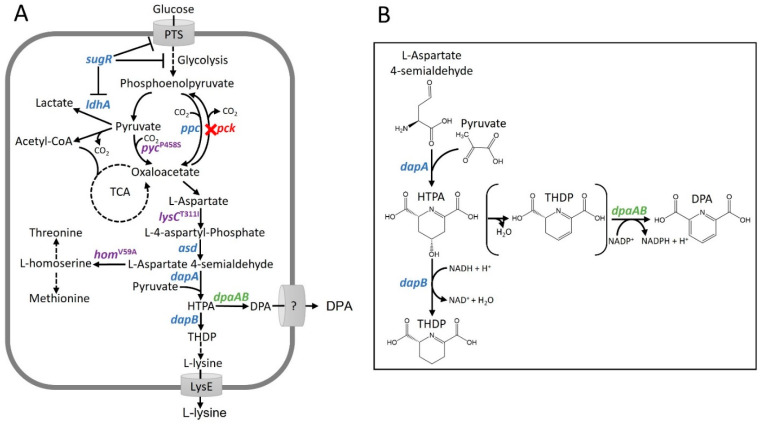
Scheme of the metabolic pathway for DPA biosynthesis in engineered *C. glutamicum* Dpa1 (**A**) and of the reactions at the branchpoint intermediate HTPA (**B**). Single catalytic steps are depicted by solid lines and multiple catalytic steps by dashed lines. It includes endogenous genes (blue) and heterologous dipicolinate synthase genes *dpaAB* from *P. sonchi* SBR5 (green). Variants of endogenous genes (purple), namely feedback-resistant aspartokinase *lysC*^T311I^, pyruvate carboxylase *pyc*^P458S^ and homoserine dehydrogenase *hom*^V59A^, increase L-aspartate conversion and anaplerosis via pyruvate carboxylase, while decreasing drain of aspartate-4-semialdehyde towards threonine and methionine biosynthesis. DPA shares the first three steps of L-lysine biosynthesis via aspartokinase (*lysC*), aspartate-semialdehyde dehydrogenase (*asd*) and HTPA synthase (*dapA*). A red cross indicates the deletion of carboxykinase gene *ppc* (red) to prevent decarboxylation of oxaloacetate. Glucose uptake is PTS (phosphotransferase system) mediated and L-lysine is exported via LysE. Brackets depict putative spontaneous dehydration of HTPA.

Biosynthesis of DPA in sporulating bacteria only requires a single committed step to convert an intermediate of the L-lysine biosynthesis pathway to DPA. For this reaction, both the oxidoreductase SpoVFA and the flavoprotein SpovFB (corresponding to the gene products of *dpaA* and *dpaB*) of dipicolinate synthase SpoVF are required for DPA synthesis by *B. subtilis* [27]. The first heterologous DPA biosynthesis resulted from overexpression of the *B. subtilis spoVFAB* operon in *Escherichia coli* [27]. Orthologous enzymes to SpoVF were later identified in *Clostridia*, but designated EftA [28]. The exact branchpoint intermediate of DPA synthesis remained controversial for a long time and was revealed as (2S, 4S)-4-hydroxy-2,3,4,5-tetrahydrodipicolinic acid (HTPA) [29] and not (S)-2,3-dihydrodipicolinate, as suggested earlier [30,31]. HTPA is synthesized by 4-hydroxy-tetrahydrodipicolinate synthase DapA (reclassified from EC 1.3.1.26 to EC 1.17.1.8) in L-lysine biosynthesis (Figure 1A).

As a branch point metabolite between L-lysine biosynthesis and DPA biosynthesis, HTPA is converted by HTPA reductase DapB towards L-lysine or to DPA by dipicolinate synthase (Figure 1A) [32]. Dipicolinate synthase DpaAB (Figure 1B) has two subunits encoded either by *spoVFAB* in *Bacilli* or by *dpaAB* in *Clostridia*, where the electron transfer protein EtfA participates in DPA biosynthesis [28]. Dipicolinate synthase catalyzes the dehydration and reduction of HTPA to DPA and a Km value of 0.776 mM HTPA for SpovFAB was determined [32]. It is possible that dehydration occurs spontaneously followed by enzyme-catalyzed reduction by dipicolinate synthase (Figure 1) [28] or that dipicolinate synthase functions as dual reductase-dehydratase similar to, e.g., TerBC in *p*-terphenyl biosynthesis [33].

Chemical production of DPA involves the oxidation of the methyl-group of 2,6-lutidine, a heterocyclic aromatic organic compound, which is isolated from coal tar and bone oil [34,35]. Albeit chemical synthesis typically allows for high titers easing product purification, there is an increasing demand for sustainable bioprocesses such as production of DPA. One challenge to establish bio-production is the natural restriction in DPA biosynthesis to the sporulation phase and its repression in vegetative cells [27]. This limitation was overcome by a promoter exchange in the natural producer *B. subtilis* combined with media optimization [36]. Introduction of *B. subtilis* sporulation genes *spoVFAB* into an *E. coli* strain that was engineered for L-lysine production allowed for DPA production by recombinant bacterium not naturally synthesizing DPA [32]. The Gram-positive soil bacterium *Corynebacterium glutamicum* naturally produces L-glutamate [37] and has been used for decades in the biotechnological industry for fermentative production of the amino acids L-glutamate and L-lysine, which reached 3.2 and 2.6 million tons, respectively [38]. Therefore, L-lysine overproducing *C. glutamicum* are ideal hosts for production of compounds that can be derived from L-lysine or intermediates of L-lysine biosynthesis. A strong metabolic engineering tool box is available for *C. glutamicum* including traditional mutagenesis and selection [39], rational strain design [40], genome reduction [41,42] and the CRISPR tools [43], including CRISPR interference [44]. Based on the strong performance of L-lysine producing *C. glutamicum* strains [38], production of L-lysine derived products has been enabled by extension of L-lysine biosynthesis. This included production of L-pipecolic acid (L-PA) [45], 4-hydroxylysine [46], cadaverine [47], 3-hydroxycadaverine [46], 5-aminovalerate (5AVA) [48,49], glutarate [22,50,51] or L-2-hydroxyglutarate [52] as well as inclusion of L-lysine into polymers such as cyanophycin [53]. L-lysine biosynthesis was intercepted in L-lysine overproducing strains to enable production of ectoine and hydroxyectoine from the intermediate aspartate-semialdehyde [54,55] or 3-aminopropionate from L-aspartate [56].

Traditional *C. glutamicum* fermentation uses phosphate as a phosphorus source and glucose as the dominant carbon source despite its competing uses in human and animal nutrition. *C. glutamicum* naturally grows with the monosaccharides glucose, fructose and ribose [57], the disaccharides sucrose and maltose, the alcohols inositol and ethanol [58,59,60,61], the organic acids pyruvate, propionate and lactate [62,63] as well as some amino acids [64]. A flexible feedstock concept has been achieved by metabolic engineering [65,66] and it enabled *C. glutamicum* access to non-native carbon sources such as starch, glycerol from fats, pentoses from lignocellulosic hydrolysates and amino sugars from shrimp waste [67]. L-lysine and derived compounds were produced from alternative carbon sources, e.g., cadaverine from starch or xylose [68,69] and ectoine from starch, xylose, arabinose, glycerol and glucosamine [55]. Typically, *C. glutamicum* co-utilizes the alternative with the native carbon sources, e.g., xylose with glucose [70], while yields remain lower than with glucose with the notable exception of sarcosine production [71]. Traditional *C. glutamicum* fermentations do not suffer from phage lysis but are operated in sterilized steel tanks to avoid microbial contamination. Sterilization is money-, time- and resource-consuming [72,73]. Non-sterile contamination-free fermentation processes are important [74] and have been realized for *B. subtilis*, *E. coli*, and *C. glutamicum* [72,73,75]. The use of phosphite instead of phosphate combined with expression of the phosphite dehydrogenase gene *ptxD* from *Pseudomonas stutzeri* allowed non-sterile production of L-lysine by *C. glutamicum* [72,75].

In this work, we describe the first *de novo* production of the aromatic dicarboxylic acid DPA by *C. glutamicum*. To lay the foundation for sustainable production, DPA was produced from the alternative carbon sources fructose, xylose, arabinose, glycerol, and starch on the one hand and, on the other hand, *ptxD* from *Pseudomonas stutzeri* was expressed for non-sterile production using phosphite instead of phosphate as source of phosphorus.

## 2. Materials and Methods

### 2.1. Bacterial Strains and Growth Conditions

The strains and plasmids used in this study are listed in (Table 1). *E. coli* DH5α [76] was used for vector construction and amplification and grown in lysogeny broth (LB) at 37 °C and 180 rpm. According to the vectors, the medium for *E. coli* was supplemented with tetracycline (10 μg·mL^−1^) and/or kanamycin (50 μg·mL^−1^), spectinomycin (100 μg·mL^−1^) or chloramphenicol (30 μg·mL^−1^).

Precultures of *C. glutamicum* were inoculated from fresh LB agar plates and cultivated in 10 or 50 mL brain-heart infusion broth (BHI) (ROTH, Karlsruhe, Germany) in 50 or 500 mL baffled flasks at 30 °C and 120 rpm on a rotary shaker. If not stated otherwise, production and growth experiments were routinely performed in 50 mL baffled flasks in 10 mL CgXII minimal medium [77] with 40 g·L^−1^ glucose at 30 °C and 120 rpm on a rotary shaker. Precultures were harvested by centrifugation (3200× *g*, 7 min) and washed once in TN buffer (50 mM Tris-HCL, 50 mM NaCl, pH 6.3) prior to the inoculum of the main cultures to an optical density (OD_600_) of 1. Growth was monitored by optical density measurements at 600 nm, using a V-1200 Spectrophotometer (VWR, Radnor, PA, USA) and an OD_600_ of 1 was determined to be equivalent to 0.25 g·L^−1^ cell dry weight (CDW).

**Table 1:** Strains and plasmids used in this work.

Strains	Relevant Characteristics	Source
*E. coli* DH5α	*F-thi-1 endA1 hsdr17*(*r-, m-*) *supE44 1lacU169* (*F80lacZ1M15*) *recA1 gyrA96*	[76]
WT	*C. glutamicum wild type,* ATCC 13032	[78]
WT(pECXT_P_syn__*ptxD*)	WT carrying (pECXT_P_syn__*ptxD*)	This study
DM1729Δ*pck*	WT with genomic modifications *pyc^P458S^ hom^V59A^ lysC^T311^*Δ*pck*	[79]
DM1729Δ*sugR*Δ*ldhA*	WT with genomic modifications *pyc^P458S^ hom^V59A^ lysC^T311^*Δ*sugR*Δ*ldhA*	[55]
DM1729Δ*pck*(pECXT_P_syn_)	DM1729Δ*pck*, carrying pECXT_P_syn_	This study
DM1729Δ*sugR*Δ*ldhA*(pECXT_P_syn_)	DM1729Δ*sugR*Δ*ldhA*, carrying pECXT_P_syn_	This study
Dpa1	DM1729Δ*pck* carrying pECXT_P_syn__*dpaAB*	This study
Dpa2	DM1729Δ*sugR*Δ*ldhA* carrying pECXT_P_syn__*dpaAB*	This study
Dpa1-pDapA	Dpa1 carrying pEKEx3_*dapA*	This study
Dpa1-DapA	DM1729Δ*pck* carrying pECXT_P_syn__*dpaAB-dapA*	This study
Dpa1(pEKEx3)	Dpa1 carrying pEKEx3	This study
Dpa1(pVWEx1)	Dpa1 carrying pVWEx1	This study
Dpa1(pVWEx1_*lysC*^T311I^)	Dpa1 carrying pVWEx1_*lysC*^T311I^	This study
Dpa1(pEKEx3*_ppc*)	Dpa1 carrying pEKEx3*_ppc*	This study
Dpa1(pEKEx3_*pyc*^P458S^)	Dpa1 carrying pEKEx3_*pyc*^P458S^	This study
Dpa1(pEKEx3*_ppc-pyc*^P458S^)	Dpa1 carrying pEKEx3*_ppc-pyc*^P458S^	This study
Dpa1(pVWEx1_*araBAD*)	Dpa1 carrying pVWEx1_*araBAD*	This study
Dpa1(pVWEx1_*xlyAB*)	Dpa1 carrying pVWEx1_*xlyAB*	This study
Dpa1(pVWEx1_*glpFKD*)	Dpa1 carrying pVWEx1_*glpFKD*	This study
Dpa1(pVWEx1_*amyA*)	Dpa1 carrying pVWEx1_*amyA*	This study
Dpa1-PtxD	DM1729Δ*pck* carrying pECXT_P_syn_*_dpaAB-ptxD*	This study
Dpa1-PtxD(pVWEx1)	Dpa1-PtxD carrying pVWEx1	This study
Dpa1-PtxD(pVWEx1_*glpFKD*)	Dpa1-PtxD carrying pVWEx1_*glpFKD*	This study
**Plasmids**		
pUC57_*amiF*-*ptxD*	Amp^R^, cloning plasmid with sequences of codon-optimized versions of *amiF* from *Helicobacter pylori* and *ptxD* from *Pseudomonas stutzeri* WM88	BioCat GmbH
pECXT99A	Tet^R^, Ptac, *lacI*^q^, pGA1 minireplicon, *C. glutamicum*/*E. coli* IPTG-inducible expression shuttle vector	[80]
pAmy	pECXT99A overexpressing *amyA* from *Streptomyces griseus* IMRU 3570	[81]
pECXT_P_syn_	Tet^R^, pECXT99A derivative for constitutive expression from promoter P_syn_	[82]
pECXT_P_syn__*ptxD*	pECXT_P_syn_ derivative for expression of codon-optimized version of *ptxD* from *Pseudomonas stutzeri* WM88	This study
pECXT_P_syn__*dpaAB*	pECXT_P_syn_ derivative for expression of *dpaAB* from *Paenibacillus sonchi* genomovar *Riograndensis* SBR5	This study
pECXT_P_syn__*dpaAB-dapA*	pECXT_P_syn_ derivative for expression of *dpaAB* from *Paenibacillus sonchi* genomovar *Riograndensis* SBR5 and *dapA* from *C. glutamicum* WT	This study
pECXT_P_syn__*dpaAB-ptxD*	pECXT_P_syn__*dpaAB* also expressing codon-harmonized *ptxD* from *Pseudomonas stutzeri* WM88	This study
pVWEx1	Tet^R^, P_tac_, *lacIq* pHM1519 OriV_Cg_ *C. glutamicum*/*E. coli* expression shuttle vector	[83]
pVWEx1_*lysC*^T311I^	pVWEx1 for overexpression of *lysC*^T311I^ from *C. glutamicum* ATCC 13032	[84]
pVWEx1_*xylAB*	pVWEx1 for overexpression of *xylA* from *Xanthomonas campestris* SCC1758 and *xylB* from *C. glutamicum* WT	[70]
pVWEx1_*araBAD*	pVWEx1 for overexpression of *araBAD* from *E. coli* MG1655	[85]
pVWEx1_*glpFKD*	pVWEx1 for overexpression of of *glpK, glpD* and *glpF* from *E. coli* MG1655	[86]
pVWEx1_*amyA*	pVWEx1 for overexpression of *amyA* from *Streptomyces griseus* IMRU3570	This work
pEKEx3	Spec^R^, P_tac_, *lacI*^q^, pBL1 OriV_Cg_, *C. glutamicum*/*E. coli* expression shuttle vector	[87]
pEKEx3_*pyc*^P458S^	pEKEx3 for overexpression of *pyc*^P458S^ from *C. glutamicum* WT	[88]
pEKEx3_*ppc*	pEKEx3 for overexpression of *ppc* from *C. glutamicum* WT	[88]
pEKEx3_*ppc-pyc*^P458S^	pEKEx3 for overexpression of *pyc*^P458S^ and *ppc* from *C. glutamicum* WT	This study
pEKEx3_*dapA*	pEKEx3 for overexpression of *dapA* from *C. glutamicum* WT	This study
pS_dCas9	Cm^R^, P_tac_, *lacI^q^,* P_tetA_, *tetR* pHM1519 OriV_Cg_ CRISPRi vector, anhydrotetracycline-inducible expression of *dCas9* from *S. pyogenes* and IPTG-inducible expression of the dCas9 handle	[89]
pS_dCas9_*dapB*14	pS_dCas9 plasmid carrying the *dapB14* sgRNA	This study
pS_dCas9_*dapB*561	pS_dCas9 plasmid carrying the *dapB561* sgRNA	This study

The cultivation with alternative carbon sources was performed at a concentration of 20 g·L^−1^. Deviating, soluble starch was added at 10 g·L^−1^ due to low solubility and 0.5 g·L^−1^ glucose was added as co-substrate to enable *C. glutamicum* to utilize starch. Phosphorous-substituted (P-substituted) CgXII contained the indicated concentration of di-sodium phosphite instead of di-potassium hydrogen phosphate and potassium di-hydrogen phosphate. Growth experiments for the determination of the sensitivity to DPA were carried out in a BioLector microcultivation system (m2p-labs, Aachen, Germany) in a volume of 1 mL CgXII minimal medium with 40 g·L^−1^ glucose in a 48-well flower plate at 30 °C, 85% humidity and a shaking frequency of 1100 rpm. A signal gain of 15 was used to follow growth by backscatter light signal at 620 nm.

According to the vectors, the medium for *C. glutamicum* was supplemented with tetracycline (5 μ·mL^−1^) and/or kanamycin (25 μ·mL^−1^), spectinomycin (100 μg·mL^−1^) or chloramphenicol (7 μg·mL^−1^). Gene expression from pVWEx1 and pEKEx3-derived plasmids was induced by the addition of 1 mM Isopropyl-β-D-1-thiogalactopyranoside (IPTG) and the CRISPRi system by the addition of 1 mM IPTG and 0.25 μg·mL^−1^ anhydrotetracycline to CgXII minimal medium at the beginning of the cultivation. 

### 2.2. Molecular Genetic Techniques and Strain Construction

DNA sequences were amplified using the respective oligonucleotides (Tables S1 and S2), and ALLin^TM^ HiFi DNA Polymerase (highQu GmbH, Kraichtal, Germany) according to the manufacturer’s protocol. DNA of *C. glutamicum* and *P. sonchi* SBR5 served as template for the amplification of *dapA* and *dpaAB* respectively, while *ptxD* was amplified from the plasmid pUC57_*amiF-ptxD* and *amyA* was amplified from pAmy [81]. A consensus ribosome binding site (RBS) sequence (GAAAGGAGGCCCTTCAG) was inserted in front of the genes *dpaAB*, *dapA* and *ptxD* via primer overhangs.

The plasmids pECXT_P_syn_ pVWEx1 and pEKEx3 were linearized by restriction with BamHI, while PstI (NEB, Frankfurt, Germany) was used to linearize pS_dCas9 [89]. Deviating, pECXT_P_syn_*_dpaAB-ptxD* was constructed by linearization of pECXT_P_syn_*_dpaAB* with XbaI (NEB, Frankfurt, Germany) for the integration of *ptxD* amplified from pECXT_P_syn_*_ptxD*. Linearized vectors were dephosphorylated (Antarctic phosphatase, New England Biolabs, Frankfurt, Germany) prior to plasmid assembly by Gibson Assembly [90]. DNA concentration was determined at 600 nm, using a V-1200 Spectrophotometer (VWR, Radnor, PA, USA).

The sequences of inserts of plasmids, constructed in this study (Table 1), were confirmed by sequencing with the respective oligonucleotides (Table S1). All oligonucleotides used for DNA amplification and sequencing were obtained from Metabion (Planegg/Steinkirchen, Germany). Plasmids were isolated using a plasmid miniprep kit (GeneJET, Thermo Fisher Scientific, Schwerte, Germany). Standard molecular genetic techniques were carried out as described previously [91]. Competent *E. coli* cells were prepared by CaCl_2_ method [91] and transformed by heat shock at 42 °C [77], whereas competent *C. glutamicum* cells were transformed by electroporation [77]. Transformants were screened by colony PCR with the respective vector-specific fw and rv verification oligonucleotides (Table S1).

### 2.3. Analytical Procedures

Extracellular amino acids, carbohydrates and DPA were quantified using a high-pressure liquid chromatography system (HPLC) (1200 series, Agilent Technologies Deutschland GmbH, Böblingen, Germany). Cell culture supernatant was taken at the indicated time points, centrifuged (20,200× *g*, 20 min) and stored at −20 °C until analysis.

Samples for amino acid analysis were derivatized with *ortho*-phthaldialdehyde (OPA) [92], and L-asparagine served as internal standard. Amino acids were separated by reversed phase HPLC, using a pre- (LiChrospher 100 RP18 EC-5µ (40 × 4 mm), CS Chromatographie Service GmbH, Langerwehe, Germany) and a main-column (LiChrospher 100 RP18 EC-5µ, 125 × 4.6 mm, CS Chromatographie Service GmbH, Langerwehe, Germany) and detected with a fluorescence detector (FLD G1321A, 1200 series, Agilent Technologies, Deutschland GmbH, Böblingen, Germany) at an excitation wavelength of 230 nm and an emission wavelength of 450 nm.

Carbohydrates and DPA were separated with an amino exchange column (Aminex, 300 × 8 mm, 10 μm particle size, 25 Å pore diameter, CS Chromatographie Service GmbH, Langerwehe, Germany) with 5 mM H_2_SO_4_ under isocratic conditions at a flow rate of 0.8 mL·min^−1^ for 30 min as described previously [85]. The substances were detected by a refractive index detector (RID G1362A, 1200 series, Agilent Technologies, Deutschland GmbH, Böblingen, Germany). The DPA and L-lysine titers obtained from starch represent those obtained from 10 g·L^−1^ plus 0.5 g·L^−1^ glucose after subtraction of titers, obtained from 0.5 g·L^−1^ glucose alone.

### 2.4. Enzymatic Assay

Cells for crude extract were grown in LB in 500 mL baffled flasks and harvested by centrifugation (20200× *g*, 7 min) at 4 °C when stationary phase was reached. All following steps were performed on ice or at 4 °C. The pellet was resuspended in reaction buffer (100 mM MOPS, pH 7), washed three times and solved in 5 mL reaction buffer for cell disruption by sonication (UP 200S, Dr. Hielscher GmbH, Teltow, Germany) for 9 min at 60% amplitude and a duty cycle of 0.5 s. Total crude protein concentrations were determined by the method of Bradford, and bovine serum standard was used as reference [93].

Oxidative PtxD activity was measured using 4 mM NAD^+^ and 13 mM di-sodium phosphite as described previously [73,94]. Absorption was followed for 3 min at 340 nm. One unit (U) of phosphite dehydrogenase activity was defined as the quantity of enzyme required to convert 1 μmol of NADH per min at 25 °C.

### 2.5. Gene Repression by CRISPRi

CRISPRi-mediated gene repression was based on the previously developed vector pS-dCas9 [89]. Two single guide RNAs (sgRNAs) were designed with the CRISPy-web-tool [95], based on the genome sequence of *C. glutamicum* WT [78]. The targets comprise a 20 bp region, homologous to the non-template strand of *dapB.* The double-stranded sgRNA inserts were generated from single-stranded oligonucleotides by an annealing oligo method, as described elsewhere for Gibson plasmid assembly with PstI restricted pS_dCas9 [89].

### 2.6. Cultivation in a Fermenter

Fermentative production was performed in a baffled bioreactor with a total volume of 7 L (NLFNLF, Bioengineering AG, Switzerland) with two six-bladed Rushton turbines. A sparger at the bottom provided a constant airflow of 5 NL·min^−1^. The stirrer rate was set to 200 rpm and increased to 350 rpm after 18 h to maintain a relative dissolved oxygen concentration above 30%. The temperature was kept at 30 °C at the headspace overpressure of 0.2 bar. The pH was kept at 7, adjusted by the automatic addition of chloric acid (2 M) and ammonia (25% (*w*/*v*)). A CgXII preculture was used to inoculate the initial working volume of 2.5 L of CgXII minimal medium, omitting MOPS buffer and containing 40 g·L^−1^ glucose to an OD_600_ of 1. To prevent foaming, 0.6 mL·L^−1^ of the antifoam agent Pluronic F-68 were added. The feed, consisting of 100 g·L^−1^ glucose and 40 g·L^−1^ (NH_4_)_2_SO_4_, was started at 41 h and 70 h when the glucose was depleted and growth had ceased, respectively. Samples were taken by an autosampler and kept at 4 °C until analysis. Titers and yields of the fermentation were related to the initial fermentation volume of 2.5 L. All values were normalized for the rise in the fermentation volume from feed addition. Final product titers and yields were calculated using the total glucose added, comprising the initial glucose concentration (40 g·L^−1^) and the feed (39 g·L^−1^, related to the initial fermentation volume of 2.5 L). 

### 2.7. Statistical Analysis

Statistical significance was determined by the unpaired Student’s *t*-tests (two-sided), using a *p*-value of < 0.001 (***) for triplicate cultivations.

## 3. Results

### 3.1. Investigating of the Suitability of C. glutamicum for DPA Production

**Figure 2:**
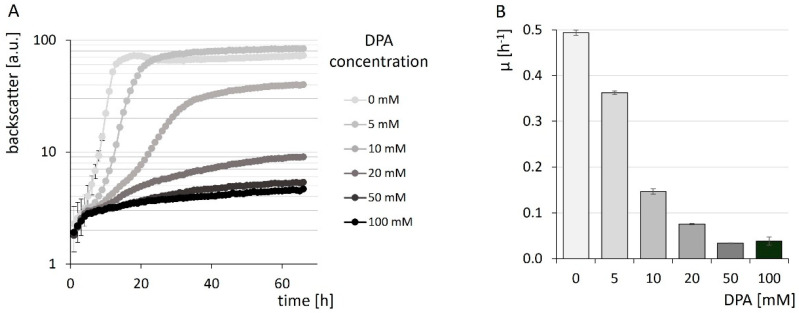
Growth curves (**A**) and maximal growth rates µ (**B**) of *C. glutamicum* WT, cultivated in the presence of 0–100 mM DPA in CgXII minimal medium, containing 40 g·L^−1^ glucose in a BioLector system. Growth was monitored by the BioLector backscatter signal, which is given as arbitrary units (a.u.) in (**A**). The maximal specific growth rates are given in h^−1^ (**B**). Values are given as means with standard deviations from triplicate cultivations.

To evaluate the suitability of *C. glutamicum* to produce DPA, growth experiments were performed to test for a potential product toxicity. *C. glutamicum* WT was grown in the BioLector microcultivation system in glucose minimal medium in the presence of 0–100 mM DPA. Growth was observed for the entire DPA concentration range tested but affected by its presence. The addition of DPA slowed growth, and biomass formation was reduced at concentrations of 10 mM (1.7 g·L^−1^) DPA or more (Figure 2A). At a DPA concentration of 9.8 ± 0.12 mM (1.63 ± 0.02 g·L^−1^) the growth rate µ was reduced to half-maximum (Figure 2B). Thus, growth of *C. glutamicum* WT in the presence of DPA is possible but was slowed by increasing DPA concentrations.

### 3.2. Engineering of L-Lysine Producing Strains for DPA Production from Glucose

Biosynthesis of DPA in *Bacilli* shares the first three steps with the L-lysine biosynthesis pathway up to the branch point intermediate HTPA. In L-lysine biosynthesis, HTPA is formed by HTPA synthase (DapA) before being immediately oxidized by dihydrodipicolinate reductase (DapB) to THDP. Instead, dehydration and reduction of HTPA by dipicolinate synthase, encoded by *dpaAB*, yields DPA [28]. Biosynthesis of DPA has not been described for *C. glutamicum*, a bacterium known for its L-lysine production potential. Inspection of the *C. glutamicum* genome [78] did not provide evidence for DPA biosynthesis genes. Thus, exogenous DPA biosynthesis genes were chosen for expression in L-lysine producing *C. glutamicum* strains.

Here, the two previously described L-lysine producing strains *C. glutamicum* DM1729Δ*sugR*Δ*ldhA* [55] and *C. glutamicum* DM1729Δ*pck* [79] were chosen as potential basis strains for the production of DPA. *C. glutamicum* strain DM1729 overproduces L-lysine due to three single nucleotide exchanges introduced into the wild type ATCC 13032, namely, *lysC*^T311I^, *pyc*^P458S^ and *hom*^V59A^. The L-lysine feedback-resistant aspartokinase, encoded by *lysC*^T311I^ [96,97] and the enhanced activity variant pyruvate carboxylase, encoded by *pyc*^P458S^ [98,99], improve the availability of the precursor oxaloacetate. The attenuated homoserine dehydrogenase (encoded by *hom*^V59A^) reduces loss of the intermediate L-aspartate-semialdehyde to biosynthesis of homoserine, L-methionine and L-threonine [100,101]. Deletion of the gluconeogenic phosphoenolpyruvate carboxykinase gene *pck* in DM1729Δ*pck* contributes to a higher oxaloacetate availability, which increased flux into L-lysine biosynthesis (Figure 1A) [102]. Strain DM1729Δ*sugR*Δ*ldhA* lacks repression control of phosphotransferase system and glycolysis genes by global transcriptional repressor SugR (Figure 1A) [103,104,105,106]. In addition, this strain does not produce lactate as unwanted by-product due to the deletion of *ldhA* that encodes fermentative NAD-dependent L-lactate dehydrogenase [107]. The strains DM1729, DM1729Δ*sugR*Δ*ldhA* and DM1729Δ*pck* were chosen deliberately to ensure availability of HTPA for DPA biosynthesis since they, unlike many other L-lysine producing strains, do not possess any modifications downstream of HTPA.

Conversion of HTPA to DPA by dipicolinate synthase is well-known in sporulating bacteria, e.g., *B. subtilis* [27]. In this work, we chose to introduce the dipicolinate synthase from *Paenibacillus sonchi* genomovar *riograndensis* SBR5 because its optimal growth temperature of 30–37 °C [108] conforms well with that of *C. glutamicum* (30 °C). The dipicolinate synthase genes *dpaAB* from *P. sonchi* SBR5 were cloned into the constitutive expression vector pECXT_P_syn_. Transformation of DM1729Δ*pck* and DM1729Δ*sugR*Δ*ldhA* with pECXT_P_syn__*dpaAB* yielded strains Dpa1 and Dpa2, respectively. The expression of dipicolinate synthase decreased the maximal growth rate and total biomass formation of both strains (Table 2).

**Figure 3:**
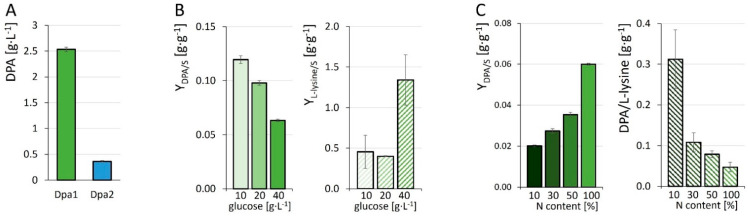
Production of DPA by Dpa1 (green) and Dpa2 (blue) (**A**), yields of DPA and L-lysine by Dpa1 at reduced glucose content (**B**) and yield of DPA and ratio of production of DPA to L-lysine at reduced nitrogen content (**C**). Cultivations were performed in CgXII containing 40 g·L^−1^ glucose (**A**,**C**) for 96 h. The glucose concentration in the medium was reduced from 40 g·L^−1^ to 20 g·L^−1^ and 10 g·L^−1^ glucose (**B**) and the concentrations of the nitrogen sources (N content) were reduced to 10%, 30% or 50% (**C**). Control strains carrying the empty vector (pECXT_P_syn_) produced no DPA (data not shown). Values are given as means with standard deviations from triplicate cultivations.

Production of DPA by the negative control strains DM1729Δ*pck*(pECXT_P_syn_) and DM1729 Δ*sugR*Δ*ldhA*(pECXT_P_syn_) could not be detected, confirming that *C. glutamicum* naturally lacks the ability of *de novo* biosynthesis of DPA. The constructed strain Dpa1 produced 2.53 ± 0.04 g·L^−1^ DPA in CgXII minimal medium containing 40 g·L^−1^ glucose in 96 h, while Dpa2 only produced 0.36 ± 0.01 g·L^−1^ (Table 2, Figure 3A). The lower DPA production by strain Dpa2 was accompanied by incomplete glucose consumption. Owing to its higher DPA production and robust growth, the following experiments were performed with Dpa1.

### 3.3. Product Inhibition May Limit DPA Production

To investigate whether production of DPA by Dpa1 was inhibited by the presence of DPA, growth and production were tested in CgXII with 40 g·L^−1^ glucose, supplemented with 1 or 2 g·L^−1^ DPA. Regardless of the concentration, µ was lower when DPA was added to glucose containing CgXII (0.03 ± 0.00 h^−1^ as compared to 0.09 ± 0.00 h^−1^). The addition of DPA perturbed growth, shown by incomplete glucose consumption in 144 h. Its presence at a concentration of 1 or 2 g·L^−1^ reduced the total biomass formation to 75% and 30% and DPA production to 62% and 43%, respectively (Table 2).

The observed sensitivity of *C. glutamicum* WT to DPA suggested that growth and growth-coupled DPA production by Dpa1 may be limited by product inhibition. Hence, lowering substrate concentrations may be a simple tool to raise the product yield Y_DPA/S_, making DPA production more efficient and sustainable due to lower resource use. To test this, Dpa1 was cultivated in CgXII with glucose concentrations reduced to 50% and 25% (20 and 10 g·L^−1^). When 25% glucose were used, Y_DPA/S_ was doubled (from 0.06 to 0.12 g·g^−1^) as compared to the regular concentration of 40 g·L^−1^ (Figure 3B). Concomitantly, the by-product L-lysine yield was reduced to one third for both 25 and 50% glucose (Figure 3 B). The cultivation with reduced glucose content was beneficial for improved product yields per substrate and the reduction of by-product formation.

Regular CgXII minimal medium contains 468 mM nitrogen in the form of urea and ammonium sulphate because it was optimized for nitrogen-demanding L-lysine production. L-lysine contains two nitrogen atoms, whereas DPA contains just one. Therefore, Dpa1 was cultivated in CgXII minimal medium containing 50%, 30% or 10% of the regular nitrogen concentration to aim for a more favorable ratio of DPA to L-lysine formation. As intended, reducing the nitrogen concentration in the medium shifted the DPA to L-lysine ratio towards DPA production and resulted in a six-fold higher DPA to L-lysine ratio with 10% nitrogen (Table 2, Figure 3C). While biomass formation was not significantly affected, the absolute DPA titers were reduced to one third (0.81 ± 0.02 g·L^−1^) with 10% nitrogen as compared to the regular nitrogen concentration in the medium (Table 2).

### 3.4. Assessment of the Effect of Increased Availability of Precursors on DPA Production

**Figure 4:**
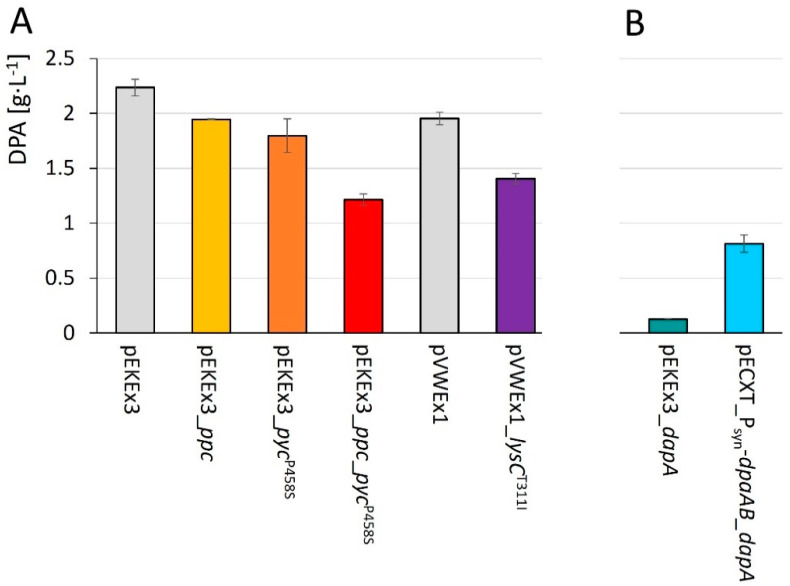
Production of DPA by Dpa1 derived strains overexpressing *ppc* (yellow), *pyc*^P458S^ (orange), *ppc* and *pyc*^P458S^ (red) or *lysC*^T311I^ (violet) from a second plasmid besides pECXT_P_syn__*dpaAB.* Control strains Dpa1(pVWEx1) and Dpa1(pEKEx3) are shown in grey (**A**). Comparison of overexpressing native *dapA* either from pEKEx3_*dapA* as a second plasmid besides pECXT_P_syn__*dpaAB* (strain Dpa1-pDapA; turquoise) or in a synthetic operon with *dpaAB* on plasmid pECXT_P_syn__*dpaAB-dapA* (strain Dpa1-DapA) (light blue) (**B**). Cultivation was performed in CgXII containing 40 g·L^−1^ glucose for 144 h (**A**) or 96 h (**B**). Concentrations are given as means with standard deviations from triplicate cultivations.

L-lysine production is known to suffer from bottlenecks regarding its initial enzyme aspartokinase [96], and regarding anaplerosis via PEP carboxylase [109] and pyruvate carboxylase [99] that is required for providing oxaloacetate as precursor. As HTPA is an intermediate of L-lysine biosynthesis is the substrate of dipicolinate synthase, pathway engineering strategies applied to increase the flux into L-lysine biosynthesis are relevant for DPA production as long as they target only reactions leading to HTPA, but not improving conversion of HTPA towards L-lysine. To investigate the impact on DPA production, the genes *lysC*^T311I^, *pyc*^P458S^, *ppc,* or *ppc* and *pyc*^P458S^ were overexpressed from an IPTG-inducible plasmid in strain Dpa1 that already carries chromosomal copies of *lysC*^T311I^ and *pyc*^P458S^. However, the resulting strains Dpa1(pVWEx1_*lysC*^T311I^), Dpa1(pEKEx3_*ppc*), Dpa1(pEKEx3_*pyc*^P458S^) and Dpa1(pEKEx3_*ppc*-*pyc*^P458S^) produced less DPA than the control strains Dpa1(pVWEx1) and Dpa1(pEKEx3). The simultaneous overexpression of both *ppc* and *pyc*^P458S^ had the most severe effect (Figure 4A). Slower growth of all strains may be due to the additional metabolic burden of the second plasmid. Thus, DPA production by strain Dpa1 could not be improved by these strategies, which indicated that DPA production was not limited by aspartokinase or anaplerosis in these strains.

Another possible limitation is the availability of HTPA, the substrate for dipicolinate synthase and the last intermediate shared with L-lysine biosynthesis. HTPA synthase (DapA) yields HTPA by condensing pyruvate with aspartate. Therefore, *dapA* was overexpressed either from a second IPTG-inducible plasmid (strain Dpa1-pDapA) or in a synthetic operon with *dpaAB* (strain Dpa1-DapA). However, biomass formation was perturbed and DPA accumulated by strains Dpa1-pDapA and Dpa1-DapA after 144 h was 11% and 36% to that of the control strains Dpa1(pEKEx3) and Dpa1, respectively (Figure 4B). Thus, HTPA availability did not limit DPA production by these strains.

Besides the target molecule DPA, Dpa1 formed 53.66 ± 12.46 g·L^−1^ L-lysine as by-product (Table 2), which reflects its background of an engineered L-lysine producer. As an alternative to the overexpression of *dapA* to improve HTPA synthesis, its drain due to conversion by HTPA reductase DapB towards L-lysine biosynthesis may be reduced. To this end, *dapB* was repressed using the CRISPRi method. Two sgRNAs sequences, targeting *dapB* proximal or distant to the translational start site, were chosen to repress *dapB.* The resulting plasmids were used to transform strain Dpa1 yielding strains Dpa1(pS_dCas9_*dapB*14) and Dpa1(pS_dCas9_*dapB*561), respectively. Strain Dpa1(pS_dCas9) served as a negative control as it expressed *dCas9* but lacked a sgRNA. CRISPRi mediated repression of *dapB* reduced L-lysine formation from 13.53 ± 0.73 g·L^−1^ to 3.53 ± 0.06 g·L^−1^ and 4.47 ± 0.12 g·L^−1^, respectively, while DPA production was increased (Table 2). Strain Dpa1(pS_dCas9_*dapB*14) grew to a significantly higher biomass concentration (*p* < 0.001; 5.46 ± 0.26 g·L^−1^ compared to 4.13 ± 0.12 g·L^−1^ by Dpa1(pS_dCas9)) and produced significantly more DPA (*p* < 0.001; 1.91 ± 0.02 g·L^−1^ compared to 1.73 ± 0.04 g·L^−1^ by Dpa1(pS_dCas9)) (Table 2). Strain Dpa1(pS_dCas9_*dapB*561) did not grow to a significantly higher biomass concentration (*p* < 0.001; 4.50 ± 0.37 g·L^−1^ compared to 4.13 ± 0.12 g·L^−1^ by Dpa1(pS_dCas9)) but produced about twice as much DPA (3.28 ± 0.12 g·L^−1^ compared to 1.73 ± 0.04 g·L^−1^ by Dpa1(pS_dCas9)) (Table 2). While the quantitative extents of CRISPRi-mediated repression of *dapB* on DPA, L-lysine and biomass concentrations varied depending on the used sgRNA targeting *dapB*, this strategy proved beneficial for improving DPA production.

### 3.5. Fermentative Production of DPA in Reactor Scale

**Figure 5:**
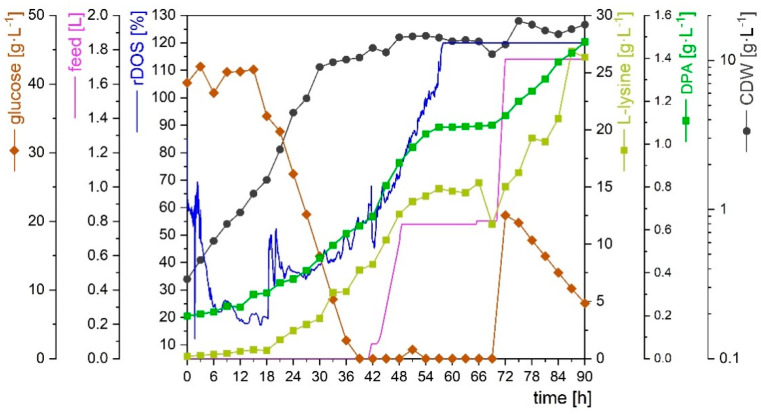
Fed-batch cultivation of Dpa1 in 2.5 L initial volume of CgXII minimal medium, containing 40 g·L^−1^ glucose for 90 h. The feed contained 100 g·L^−1^ glucose and 40 g·L^−1^ ammonium sulphate and is depicted by the purple line. The relative dissolved oxygen concentration (rDOs) is shown (blue line). Glucose (brown), L-lysine (light green), DPA (green) and biomass (black) concentrations are indicated by symbols and lines. All concentrations were calculated to the initial fermentation volume.

In order to test the robustness of DPA production, a lab-scale fed-batch fermentation of Dpa1 was performed in a 7 L bioreactor using 2.5 L CgXII minimal medium containing 40 g·L^−1^ glucose as seed medium. When glucose was depleted or cell growth had ceased, the addition of a total of 1.75 L feed, containing 100 g·L^−1^ glucose and 40 g·L^−1^ ammonium sulphate, was started manually. During the batch phase, biomass formation was twice as high as in shake flask cultivations, reaching 10.1 g·L^−1^ at 39 h (Figure 5) (as compared to 4.48 ± 0.51 (Table 2)). At 41 h, 0.75 L of feed medium were added. Biomass formation increased little, but production of both DPA and L-lysine continued (Figure 5). At 70 h, another 1 L of feed medium was added. DPA and L-lysine production resumed and continued until cultivation end at 90 h (Figure 5). In this bioreactor culture, DPA was produced to final titer of 1.47 g·L^−1^ at a yield on glucose of 0.0135 g·g^−1^ with a volumetric productivity of 0.016 g·L^−1^·h^−1^.

### 3.6. Establishing DPA Production from Alternative Carbon Sources

**Figure 6:**
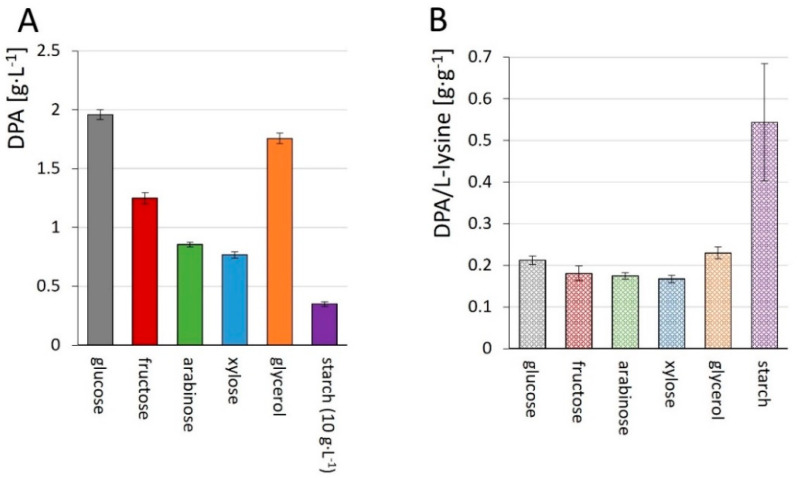
Production of DPA (**A**) and ratio of production of DPA to L-lysine (**B**) by (engineered) Dpa1 from glucose, arabinose, xylose, glycerol and starch with glucose as co-substrate. Cultivation was performed in CgXII containing 20 g·L^−1^ (10 g·L^−1^ for starch) of the respective carbon source for 72 h. Concentrations are given as means with standard deviations from triplicate cultivations.

Besides glucose, amino acid production with *C. glutamicum* uses fructose-containing molasses or second generation feedstocks [66]. Fructose is a natural source of carbon and energy for *C. glutamicum*, while metabolic engineering was required for access to the polymer starch as well as to monomeric compounds of second-generation feedstocks such as glycerol, arabinose and xylose [65]. In this regard, Dpa1 was grown on fructose and established metabolic engineering strategies were applied to allow DPA production from glycerol, xylose, arabinose, and starch. The carbon sources fructose, glycerol, arabinose, and xylose were added in a concentration of 20 g·L^−1^ and starch was added at 10 g·L^−1^. In all cases, DPA production was observed. DPA yields attained from fructose, xylose, arabinose, glycerol, and starch did not reach those from glucose, and L-lysine titers surpassed those of the target compound DPA by two to six times (Table 2, Figure 6A).

The cultivation with starch instead of glucose resulted in a more favorable DPA to L-lysine ratio (Figure 6B). Thus, DPA production from a broad spectrum of feedstocks was demonstrated.

### 3.7. Exploiting Phosphite Dehydrogenase to Establish Non-Sterile DPA Production

With the aim of establishing non-sterile production of DPA, a codon-harmonized version of the *ptxD* gene from *P. stutzeri* was expressed in *C. glutamicum* WT and the specific phosphite dehydrogenase activity was determined in crude extracts. While noactivity was detected (<0.005 U·mg^−1^) for the empty vector carrying control strains, 0.023 ± 0.002 U·mg^−1^ were determined for strain WT(pECXT_P_syn__*ptxD*), thus, demonstrating functional expression of *ptxD* in *C. glutamicum*. Indeed, only WT(pECXT_P_syn__*ptxD*) could grow with phosphite as the sole source of phosphorus (Figure S1A). In the regular CgXII minimal medium (phosphate as phosphorous source, no tetracycline), the tetracycline resistance-mediating plasmid was lost after 17 serial dilutions into fresh CgXII liquid medium, since no tetracycline resistant colonies were found upon plating on LB-tetracycline agar plates but rather on LB plates without tetracycline. When phosphate in liquid CgXII minimal medium was replaced by phosphite, the plasmid pECXT_P_syn__*ptxD* was maintained, even in the absence of tetracycline (Figure S1B). This confirmed that phosphite and *ptxD* expression can be used as a selectable trait under non-sterile growth conditions.

For co-expression with the dipicolinate synthase genes *dpaAB* in a synthetic operon, the plasmid pECXT_P_syn__*dpaAB-ptxD* was constructed and introduced into DM1729Δ*pck*, resulting in strain Dpa1-PtxD. The phosphorous sources in CgXII minimal medium were replaced with the equivalent amount (13 mM) of di-sodium phosphite. Dpa1, lacking phosphite dehydrogenase activity, did not grow with phosphite as a sole phosphorous source (Figure S2), while Dpa1-PtxD reached 45% of the biomass of Dpa1 from 40 g·L^−1^ glucose (Table 2). The yield of DPA by Dpa1-PtxD from glucose with phosphite (0.04 g·g^−1^) corresponded to approximately 60% of the yield by Dpa1 and regular CgXII minimal medium (Table 2). Thus, DPA production using phosphite is feasible and the aptitude of the utilization of phosphite as the sole source of phosphorus as selectable trait for DPA production was demonstrated.

Beyond overriding the requirement of antibiotics, it would be preferable to replace glucose with a second-generation feedstock for DPA production. On this account, the substrate spectrum of Dpa1-PtxD was broadened to glycerol, resulting in strain Dpa1-PtxD(pVWEx1_*glpFKD*).

In P-substituted CgXII, containing 13 mM phosphite, 1 mM IPTG and 40 g·L^−1^ glycerol with the omission of antibiotics, this strain achieved a similar concentration (1.64 ± 0.07 g·L^−1^) and yield (0.04 ± 0.00 g·g^−1^) as Dpa1-PtxD in P-substituted CgXII from glucose (Table 2), whereas the control strain Dpa1-PtxD(pVWEx) did not grow with glycerol as sole carbon source (Figure S3).

## 4. Discussion

In this study, we established the first *de novo* production of DPA by *C. glutamicum* from glucose and from alternative carbon sources. Moreover, utilization of phosphite instead of phosphate rendered the strain compatible with non-sterile DPA production, and we obtained the proof-of-concept of the process robustness for fermentative DPA production in fed-batch bioreactor cultivation.

Endospores of *Bacilli* and *Clostridia* contain little of the secondary metabolite DPA and sensitive methods (gold nanoparticles and nanoclusters for fluorescence detection) were developed to detect DPA [110,111,112]. Gram-scale DPA production in *B. subtilis* required addition of glutamate as a precursor to three-fold higher concentrations than the final product titer [36]. DPA production from glucose without the requirement to add an amino acid as a precursor was first implemented in recombinant *E. coli.* It reached a concentration of 4.7 g·L^−1^ DPA with a yield of about 0.11 g·g^−1^ and an overall volumetric productivity of 65 mg·L^−1^·h^−1^ [32]. The *C. glutamicum* strain constructed here produced 2.5 g·L^−1^ DPA with a yield on glucose of about 0.06 g·g^−1^ and an overall volumetric productivity of 26 mg·L^−1^·h^−1^. These titers are considerably lower than the L-lysine titers of up to 100 g·L^−1^ obtained with *E. coli* and *C. glutamicum* L-lysine producing strains [38]. Production of L-lysine-derived products capitalizes on this fact and titers for cadaverine [47] or L-PA [45] are very high, as well. Product titers are lower the longer the extension of L-lysine biosynthesis is, e.g., two or three reaction steps to 5AVA [48,113], five steps to glutarate [22] or six steps to L-2-hydroxy-glutarate [52], with a product titer of 3.5 g·L^−1^ obtained in the latter example. Similarly, interception of L-lysine biosynthesis by converting the intermediate aspartate-semialdehyde to ectoine [114] or of the intermediate L-aspartate to 3-aminopropionate [56] led to high product titers (65 g·L^−1^ and 32.3 g·L^−1^, respectively).

Overexpression of genes that have been used in L-lysine production, such as the alleles that improve aspartokinase (*lysC*^T311I^) and pyruvate carboxylase (*pyc*^P458S^) activity [115,116,117], did not increase DPA production in this work, indicating that flux into the L-lysine pathway andoxaloacetate availability is not limiting DPA production. Moreover, the availability of HTPA, the substrate for dipicolinate synthase, does not limit DPA production in the observed concentration range, as shown by overexpression of the HTPA synthase gene that did not improve DPA titers, in contrast to previous studies [118,119]. The low DPA product titers indicate that the one-step conversion of the L-lysine biosynthesis intermediate HTPA to DPA is a major limitation. The well-studied dipicolinate synthase from *B. subtilis* [32] may be an alternative to the respective enzyme from *P. sonchi* SBR5 used in this study. Little is known about dipicolinate synthases from *paenibacilli* [120], and screening for alternative sources of dipicolinate synthases may help to improve DPA production. In this respect, it has to be noted that the Km value of dipicolinate synthase from *B. subtilis* of 0.776 mM [32] is comparable to that of DapB (0.529 mM) in *E. coli* [121], the L-lysine biosynthesis enzyme competing for the shared substrate HTPA. Thus, dipicolinate synthases with higher affinities may prove helpful for increasing DPA production.

Improving HTPA supply for dipicolinate synthase by overexpression of the gene for HTPA synthesis *dapA* did not enhance DPA production but reduced the growth rate. Previously, overexpression of native *dapA* in *C. glutamicum* increased the flux towards the L-lysine pathway while lowering the flux into the competing homoserine pathway. However, this entailed a reduced growth rate and elevated L-valine, and L-alanine formation [118,122]. Therefore, the maintenance of the highly sensitive flux balance at this strategic branchpoint must be considered but fine-tuning and adjustment of *dpaA* expression levels may prove beneficial for DPA production. At the same time, *dapB* constitutes a promising target to decrease the drain of HTPA into L-lysine biosynthesis by knockdown of the gene. CRISPRi-mediated repression of *dapB* reduced L-lysine formation and increased the DPA titer and yield. Thus, decreased utilization of HTPA towards lysine biosynthesis by DapB upon CRISPRi targeting of its gene was superior to increased HTPA biosynthesis as consequence of *dapA* overexpression. This may be due to the fact that Km values for HTPA are very low, at around 0.005 mM for bacterial DapB enzymes [123]. Km values of DPA synthase for HTPA have not been described. It has to be noted that during sporulation of *B. subtilis*, RNA polymerase sigma factor SigK is promoting transcription of *dpaAB* and *dapA*; *dapB* is not part of the SigK sigmulon [124]. Thus, this transcriptional regulatory pattern of SigK-dependent expression of *dpaAB* and *dapA* in the mother cell ensures that HTPA is synthesized for conversion to DPA but is not used by DapB in L-lysine biosynthesis. Notably, DPA is transported from the mother cell to the forespore, crossing two membranes, i.e., the outer and inner forespore membranes. The transporter SpoVV is located in the outer forespore membrane [125], and SpoVA in the inner forespore membrane [126]. Expression of *spoVV* and *spoVA* is orchestrated with SigE-dependent transcription of *spoVV* during engulfment prior to SigG-dependent expression of *spoVA* after completion of engulfment, finally followed by SigK-dependent expression of *dpaAB* and *dapA* in the mother cell [125].

Transport engineering is a valid metabolic engineering strategy [127]. Production of L-lysine derived products benefitted from deletion of *lysE*, which codes for the export system for L-lysine, L-arginine and L-citrulline [128,129], since loss of L-lysine is precluded [54,130]. However, the growth rate may be decreased if the L-lysine biosynthesis pathway is clogged upon deletion of *lysE* [130]. This was also observed when L-lysine biosynthesis was intercepted for ectoine production and *lysE* was deleted [55]. This growth impediment indicates that deletion of *lysE* is only beneficial if production of these compounds is already almost as high as L-lysine production by the parent strains. In *C. glutamicum*, DPA production shown here revealed that DPA is exported, but the export system is not known. There are no homologs of *spoVV* and *spoVA* (s. above) encoded in the *C. glutamicum* genome. Transport engineering by deregulated expression of *spoVV* during vegetative growth led to secretion of about 65 mg·L^−1^ DPA [125].

The decreased production with the presence of increased concentrations of DPA in the cultivation medium suggests that inherent characteristics of DPA may perturb growth of the production host, e.g., divalent ion chelating. The enzyme 3-deoxy-D-arabino-heptulosonate 7-phosphate synthase of the shikimate pathway from *B. subtilis* is inhibited by DPA as it contains iron and zinc ions essential for activity [131]. The slight inhibitory effect of DPA on growth of *C. glutamicum* WT is not yet understood, however, the structure of its 3-deoxy-D-arabino-heptulosonate 7-phosphate synthase also contains a divalent cation, namely Mn^2+^ [132]. In recent years, adaptive laboratory evolution has emerged as an excellent approach to select for improved growth, tolerance and production [133,134,135,136]. Tolerance of *C. glutamicum* to methanol [137,138], anthranilate [139] and indole [140] was improved by this strategy that may be applicable to increasing tolerance to DPA. When engineered such that DPA production would be required for growth using a metabolic engineering strategy known as flux enforcement, adaptive laboratory evolution can be employed to enhanced production, as has been shown for glutarate production by *C. glutamicum* [51]. In addition, media optimization, e.g., with regard to the C/N substrate ratio [141] or concentrations of supplements [36], may improve DPA production.

DPA production was also shown from alternative carbon sources. Production from xylose, arabinose and fructose was reduced considerably as compared to glucose. In the case of fructose, the titer was reduced to 65% of that from glucose (Figure 6A). Similarly, L-lysine production from fructose is lower than from glucose [116], since the carbon flux via the oxidative pentose phosphate pathway is lower on fructose than on glucose, resulting in lower NADPH availability [142]. The biosyntheses of L-lysine and DPA require four and three NADPH molecules per product molecule, respectively. This is also the reason why less L-lysine and DPA is formed from the carbon sources arabinose and xylose as these pentose sugars enter the pentose phosphate pathway without concomitant NADPH formation [70,143]. By contrast, the more reduced glycerol supported higher production of DPA (Table 2, Figure 6), putrescine [144] and L-lysine [86]. Thus, DPA production from glycerol and pentoses that can be generated from fats and second-generation feedstocks such as lignocellulose or agricultural waste materials, is possible. Recent examples for the production based on agricultural sidestreams by recombinant *C. glutamicum* are *cis*, *cis*-muconic acid from lignin [145], 2-hydroxy-glutarate and 5AVA from rice straw hydrolysate or wheat sidestream [52,146,147].

Biorefinery concepts also operate using microbial consortia that may be designed for that purpose [148]. Synthetic consortia with *C. glutamicum* have been used to access chitin and starch with mutually dependent substrate converter and producer strains [67,149]. In biorefinery concepts, the omission of antibiotics in a non-sterile environment may be particularly beneficial to reduce media complexity and cost. DPA production exploiting *ptxD* and phosphite as selective trait was shown here and is expected to be compatible with second-generation feedstock-based production of DPA in non-sterile processes.

While DPA production was stable at the 2.5 L bioreactor scale, more substrate was converted to biomass and less to DPA when compared to the shake flask cultures. It is tempting to speculate that the cation chelating effect of DPA on growth in the bioreactor is different than in shake flasks. Alternatively, or in addition, changed fluxes in the lysine pathway, which also provides *meso*-diaminopimelic acid for cell wall peptidoglycan biosynthesis, may affect the cell wall strength and, thus, have different consequences under the different mechanical forces of shake flasks and bioreactors. The molecular reason(s) and possible adaptive regulatory mechanisms remain to be identified.

This study established a proof-of-principle of *de novo* production of DPA by *C. glutamicum* from glucose and from alternative carbon sources compatible with non-sterile conditions. To achieve commercially relevant titers, yields and productivities, the discussion above may guide the further improvement of strain and process.

## Figures and Tables

**Table 2:** Growth, DPA and L-lysine production values of engineered DPA producing strains (and control strains). Cultivation was performed in shake flasks using CgXII containing 40 g·L^−1^ glucose as regular medium and cultivation for 96 h. Deviating medium composition is indicated. Cultivation of Dpa1 and Dpa1-derived strains on alternative carbon sources was performed in CgXII containing 20 g·L^−1^ fructose, xylose, arabinose, or glycerol, or 10 g·L^−1^ starch for 72 h. Values are given as means with standard deviations from triplicate cultivations.

Strain	Medium Composition	Biomass (g·L^−1^)	DPA (g·L^−1^)	Yield Y_DPA/S_ (g·g^−1^)	L-Lysine (g·L^−1^)
Dpa1	regular	4.48 ± 0. 51	2.53 ± 0.04	0.06 ± 0.00	53.66 ± 12.46
DM1729Δ*pck* pECXT_P_syn_	regular	4.82 ± 0.16	<0.01	<0.01	n.d.
Dpa2	regular	1.91 ± 0.17	0.36 ± 0.01	0.01 ± 0.00	n.d.
DM1729Δ*sugR*Δ*ldhA* pECXT_P_syn_	regular	3.47 ± 0.24	<0.01	<0.01	n.d.
Dpa1	50% C-source	2.63 ± 0.01	1.96 ± 0.04	0.10 ± 0.00	9.11 ± 0.23
Dpa1	25% C-source	1.56 ± 0.06	1.20 ± 0.04	0.12 ± 0.00	4.00 ± 0.10
Dpa1	50% N-sources	3.91 ± 0.34	1.41 ± 0.05	0.08 ± 0.00	17.93 ± 1.26
Dpa1	30% N-sources	5.15 ± 0.28	1.10 ± 0.04	0.11 ± 0.00	10.15 ± 1.80
Dpa1	10% N-sources	5.32 ± 0.67	0.81 ± 0.02	0.31 ± 0.00	2.58 ± 0.52
Dpa1	+1 g·L^−1^ DPA	3.33 ± 0.09	1.57 ± 0.07	0.04 ± 0.00	n.d.
Dpa1	+3 g·L^−1^ DPA	1.34 ± 0.09	1.08 ± 0.01	0.03 ± 0.00	n.d.
Dpa1(pS_dCas9)	regular	4.13 ± 0.12	1.73 ± 0.04	0.04 ± 0.00	13.53 ± 0.73
Dpa1(pS_dCas9_ *dapB*14)	regular	5.46 ± 0.26	1.91 ± 0.02	0.05 ± 0.00	3.53 ± 0.06
Dpa1(pS_dCas9_ *dapB*561)	regular	4.50 ± 0.37	3.28 ± 0.12	0.08 ± 0.00	4.47 ± 0.12
Dpa1	20 g·L^−1^ fructose	3.22 ± 0.26	1.25 ± 0.05	0.06 ± 0.00	6.91 ± 0.42
Dpa1(pVWEx1-*glpFKD*)	20 g·L^−1^ glycerol	3.89 ± 0.17	1.76 ± 0.04	0.09 ± 0.00	7.65 ± 0.29
Dpa1(pVWEx1-*xylAB*)	20 g·L^−1^ xylose	3.23 ± 0.31	0.77 ± 0.03	0.04 ± 0.00	4.60 ± 0.09
Dpa1(pVWEx1-*araBAD*)	20 g·L^−1^ arabinose	2.77 ± 0.08	0.85 ± 0.02	0.04 ± 0.00	4.89 ± 0.11
Dpa1(pVWEx1-*amyA*)	10 g·L^−1^ starch	2.10 ± 0.12	0.35 ± 0.02	0.04 ± 0.00	0.65 ± 0.13
Dpa1-PtxD	CgXII with phosphite as sole P source; 40 g·L^−1^ glucose	2.03 ± 0.15	1.46 ± 0.03	0.04 ± 0.00	n.d.
Dpa1-PtxD(pVWEx1-*glpFKD*)	CgXII with phosphite as sole P source; 40 g·L^−1^ glycerol	3.92 ± 0.31	1.64 ± 0.07	0.04 ± 0.00	n.d.

## Data Availability

All data are present in the manuscript and its Supplement.

## Supplementary Materials

The following supporting information can be downloaded at: https://www.mdpi.com/article/10.3390/microorganisms10040730/s1, Figure S1: Growth curves of *C. glutamicum* (pECXT_P_syn__*ptxD*) and control strain *C. glutamicum* (pECXT_P_syn_); Figure S2. Growth curves of *C. glutamicum* Dpa1-PtxD and control strain Dpa1; Figure S3. Growth curves of *C. glutamicum* Dpa1-PtxD(pVWEx1_*glpFDK*) control strain Dpa1-PtxD(pVWEx1); Table S1. Oligonucleotides used in this work; Table S2. DNA sequences used in this work.
