# Supplementary material for: Metabolic Engineering of Corynebacterium glutamicum for Sustainable Production of the Aromatic Dicarboxylic Acid Dipicolinic Acid
Source: Microorganisms. 2022 Mar 29;10(4):730. doi: 10.3390/microorganisms10040730 (PMC9024752; doi:10.3390/microorganisms10040730)
Supplement: Supplementary file 1 [file microorganisms-10-00730-s001.zip › microorganisms-1658572-supplementary.pdf]

Supplementary material to

Metabolic engineering of *Corynebacterium glutamicum* for sustainable production of the aromatic dicarboxylic acid dipicolinic acid

Lynn S. Schwardmann[1], Florian Meyer[1] oder Thomas Schäffer[2], Aron K. Dransfeld[1], Volker F. Wendisch[1*]

[1] Genetics of Prokaryotes, Faculty of Biology & CeBiTec, Bielefeld University, Universitätsstr. 25, 33615 Bielefeld, Germany; l.schwardmann@uni-bielefeld.de (L.S.S.); aron.dransfeld@uni-bielefeld.de (A.K.D.)
* Correspondence: Volker.wendisch@uni-bielefeld.de; Tel.: +49-521-106-5611

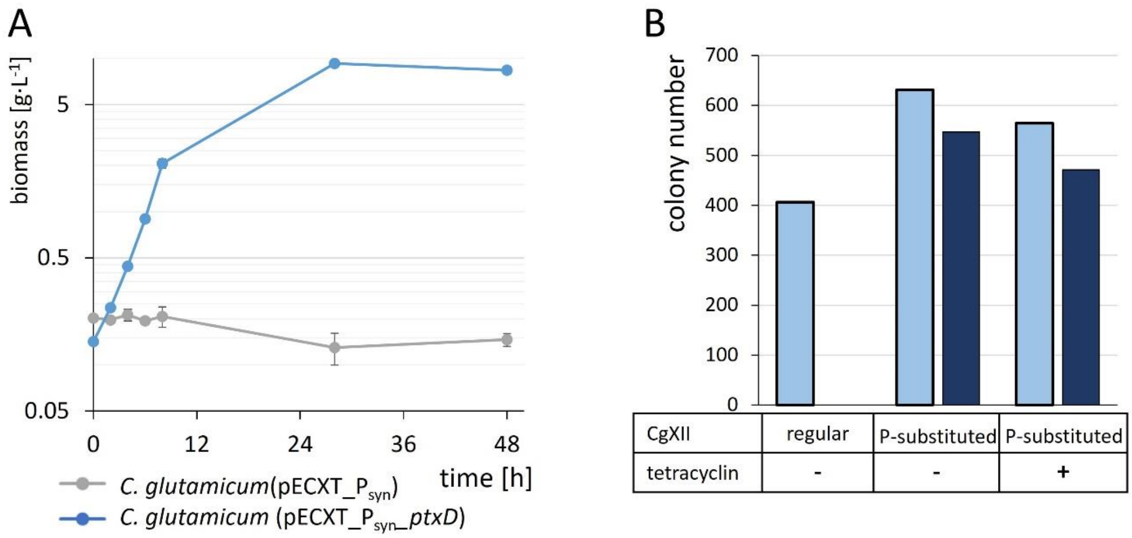

Figure S1. Growth curves of *C. glutamicum*(pECXT_P_{syn}_*ptxD)* and control strain *C. glutamicum*(pECXT_P_{syn}). The strain expressing phosphite dehydrogenase from *Pseudomonas stutzeri* WM88 (blue) and the empty vector control strain (grey) were cultivated in P-substituted CgXII minimal medium, containing 40 g·L^{-1} glucose and 13 mM di-sodium phosphite as sole phosphorous source. Values are given as means with standard deviations from technical triplicates (A). Colony numbers of *C. glutamicum*(pECXT_P_{syn}_*ptxD)* on LB (light blue) or LB-tetracyclin agar plates, after cultivation for 17 serial dilutions in regular (phosphate as phosphorous source, no tetracycline) or in P-substituted CgXII minimal medium (13 mM di-sodium phosphite as phosphorous source) with or without tetracyclin. After cultivation in P-substituted CgXII minimal medium with or without the addition of tetracyclin, colony numbers were similar, whereas the plasmid was lost after cultivation in regular CgXII without tetracyclin, demonstrating the aptitutude of *ptxD* expression as selectable trait under non-sterile growth conditions (B).

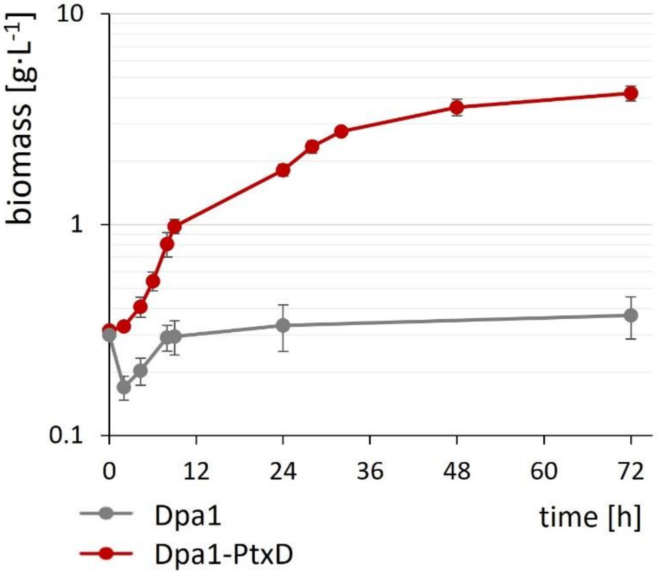

Figure S2. Growth curves of *C. glutamicum* Dpa1-PtxD and control strain Dpa1. The DPA producing strain expressing phosphite dehydrogenase from *Pseudomonas stutzeri* WM88 (red) and the control strain (grey) were cultivated in P-substituted CgXII minimal medium, containing 40 g·L-1 glucose and 13 mM di-sodium phosphite as sole phosphorous source. Values are given as means with standard deviations from technical triplicates.

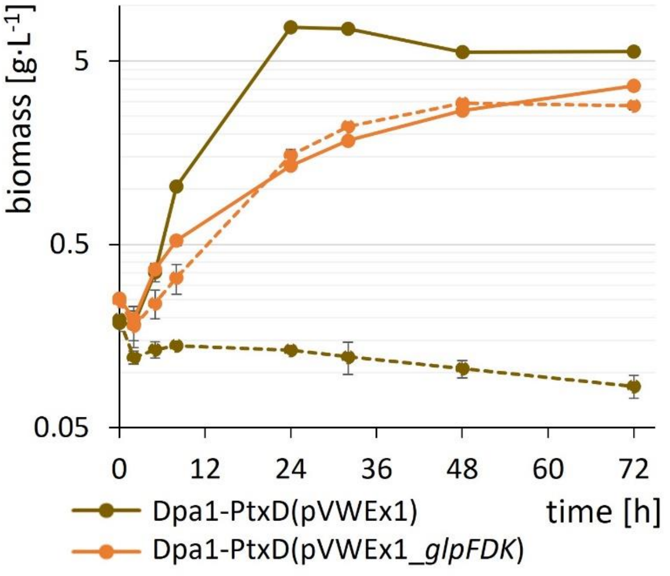

Figure S3. Growth curves of *C. glutamicum* Dpa1-PtxD(pVWEx1_*glpFDK*) control strain Dpa1-PtxD(pVWEx1). The DPA producing strain expressing phosphite dehydrogenase from *Pseudomonas stutzeri* WM88 (orange) and the control strain (brown) were cultivated in P-substituted CgXII minimal medium, containing 40 g·L-1 glucose (solid lines) or 40 g·L-1 glycerol (stacked lines) and 13 mM di-sodium phosphite as sole phosphorous source. Values are given as means with standard deviations from technical triplicates.

Table S1. Oligonucleotides used in this work.

Oligonucleotide	Sequence (5'-3')	Function
ptxD-fw-XbaI	GATTATCGAACGGTTTCATTCAGCATAGCTGAAAG GAGGCCCTTCAGATGCTGC	Amplification of *ptxD*
ptxD-rv-XbaI	GCTTGCATGCCTGCAGGTCGACTTTAATCCGCAGCT GGATTCGCC	Amplification of *ptxD*
ptxD-s1	CTTCAGGGCACAGCCGATAAC	Verification of *ptxD* sequence
ptxD-s2	GGAGGGTATCCGCGTTCAG	Verification of *ptxD* sequence
dpaAB-fw-XbaI	CAAAGGCGAATCCAGCTGCGGATTAACTCTGAAAG GAGGCCCTTCAGATGCTTAC	Amplification of *dpaAB* for coexpression with *ptxD*
dpaAB-rv-XbaI	GCTTGCATGCCTGCAGGTCGACTCTATGCTGAATGA AACCGTTCG	Amplification of *dpaAB* for coexpression with *ptxD*
dpaAB-fw	GGTTCCATGGAATTCGAGCTCGGTACCCGGGGAAA GGAGGCCCTTCAGATGCTTACTGGCATCAGGATCG	Amplification of *dpaAB*
dpaAB-rv	GCTTGCATGCCTGCAGGTCGACTCTAGAGCTATGCT GAATGAAACCGTTCG	Amplification of *dpaAB*
dpaAB-rv-*dapA*	CTTAGCTGTTAAACCTGTGCTCATCTGAAGGGCCTC CTTTCCTATGCTGAATGAAACCGTTCG	Amplification of *dpaAB* for coexpression with *dapA*
dpaAB-*dapA*-fw	CGAACGGTTTCATTCAGCATAGGAAAGGAGGCCCT TCAGATGAGCACAGGTTTAACAGCTAAG	Amplification of *dapA* for coexpression with *dpaAB*
dpaA-s	CAGGGACTCGGTTCCAACG	Verification of *dapA* sequence
dapA-rv	GCTTGCATGCCTGCAGGTCGACTCTAGAGTTATAGA ACTCCAGCTTTTTCATGTCTTCTC	Verification of *dapA* sequence
pECXT_P_{syn}-fw	TCAGTGAGCGAGGAAGC	Verification of pECXT_P_{syn} plasmid sequences and transformants
pECXT99A_rv	TACTGCCGCCAGGCAAATTC	Verification of pECXT_P_{syn} plasmid sequences and transformants
amyA-fw	GAGCTCGGTACCCGGGGATCTCAGCCGCGCCAGGT GTCGT	Amplification of *amyA*
amyA-rv	GAGCTCGGTACCCGGGGATCTCAGCCGCGCCAGGT GTCGT	Amplification of *amyA*
dapB-sg561-fw	GCCAAGCTTGCATGCCTGCAGCCCATCACAGCGTT AGGAG	Generation of sgRNA for *dapB*561 knockdown
dapB-sg561-rv	GCTATTTCTAGCTCTAAAACCTCCTAACGCTGTGAT GGGC	Generation of sgRNA for *dapB*561 knockdown
dapB-sg14-fw	GCCAAGCTTGCATGCCTGCAAATGCTCAAGTCCTA CGACT	Generation of sgRNA for *dapB*14 knockdown
dapB-sg14-rv	GCTATTTCTAGCTCTAAAACAGTCGTAGGACTTGAG CATT	Generation of sgRNA for *dapB*14 knockdown
dCas9_s-fw	CCGCTTCTGCGTTCTGATTTAATCT	Verification of pS_dCas9 plasmid sequences and transformants
dCas9_s-rv	GTATGGCTGTGCAGGTCGTAAA	Verification of pS_dCas9 plasmid sequences and transformants

Table S2. DNA sequences used in this work.

DNA sequence of the used version of the *dpaAB* gene from *Paenibacillus sonchi* genomovar *riograndensis* SBR5 for construction of pECXT_P_{syn}_*dpaAB*.

ATGCTTACTGGCATCAGGATCGTGTTCCTGGGCGGGGACGCGAGACAGATTGAAGTGATTCGGAAATGTGTGGAAATGGATG
CTACGGTAAGCGCTGCCGGGTTCGACAAGTGGGATGCCCCAAGCCCGGGGGTAAGCCTGGAACAAATGTCGGCAGAGCTGCT
GAGCCGCGCGGATGTGCTGGTGCTGCCAACGGTGGGGTGTGATGATGAAGGCAATATCAGTGCCCTCTTTTCAACGGAGCGC
CTGCAGCTGCTGGAGGAACATATCGCCGCGCTTCCGCCAAGCTGTATGGTCTATACCGGCATGGCCAAAAGCTACTTGCGCGG
CCTGTGCGACAAATATTCACTGAAGCTGGTGGAGCTGCTGAGCAGGGACGATGTGGCGATCTACAACTCCATCCCTACAGCAG
AGGGAGCATTGGTTATGGCCATTCAGAATACGGATTTTACAATCCATGGCTCTACCTCAATGGTTCTGGGCATGGGCAGAACAG
GGTTTACCATGGCCAGAGTGCTGCAGGGACTCGGTTCCAACGTGAAGGTGGGAGTAAGAAAACAGGAGCATTACGCACGGGC
CGAGGAGATGGGCTGGAAGCCCTTTATGACTGGAGAGCTGGTGGCGCATGCGCCGGAGGCGGATCTTATTTTCAACACCATCC
CTAGTATGATTATCACCGCACAAGTGTTATCGCGTCTTCCCAGACACTGTGTAATTATCGATCTGGCTTCCGCCCCGGGCGGGTG
TGATTTCCGCTATGCGGAGAAACGCGGGATCAAGGCCATGCTGGCACCGGGACTGCCCGGAGTCGTTGCTCCCAAAAGCGCCG
GAATTATTATGGCGGGCGCGCTGGTACAGTCGATATCGGACGAGACTTTTAACAGGGGGGACGTTTAATGGATTGGCATGGA
AAAACAGTAGGTTATGCGGTGACCGGCTCACACTGCACGTTAGCCGAGGTTATGCCGCAGATTCAGCGGTTTATGGAGGGCGG
AGCCAATGTGGTGCCGATTGTGTCCGCATCCGTGCTGAATACGGACACCCGCTTTGGCACATCGGAAAATTGGCTAAAACAGTT
GAAAGAAATAACAGGGAATGATATCATTTCTACAATTGTTGAAGCGGAACCGCTGGGTCCTTCCAAGCTGCTGGATGTGCTGA
CTATTGCACCCTGCACGGGGAATACGACAAGTAAATTGGCTAACGCCATGACCGACAGCCCCGTGCTGATGGCCGCCAAAGCG
CAGCTGCGCAACAGCCGTCCGCTGGTGCTGGCAATCTCCACCAATGATGGACTTGGCCTGAATGCGGCGAATATCGCGAAGCT
CCTGGTTGCGAAGAACATTTATTTTGTTCCGTTCGGCCAGGATAATCCGCAGGGCAAGCCGAATTCGCTTGTGGCCCAGATGGA
CCTCATTCCCGAAGCCTGCTTTGCGGCTTTGCAGGGCCAACAGCTGCAGCCGATGATTATCGAACGGTTTCATTAG

DNA sequence of codon-optimized version of the *ptxD* gene from *Pseudomonas stutzeri* WM88 for construction of pECXT_P_{syn}_*ptxD*.

ATGCTGCCAAAGCTCGTTATCACCCACCGCGTTCACGAAGAAATTTTGCAGCTGCTCGCACCACACTGCGAACTGATCACCAAC
CAAACCGATTCTACCCTCACCCGTGAAGAAATTTTGCGCCGTTGTCGCGATGCACAGGCCATGATGGCTTTCATGCCAGATCGT
GTGGATGCAGATTTCCTGCAAGCCTGCCCAGAACTCCGCGTTATCGGCTGTGCCCTGAAGGGTTTCGATAATTTCGATGTTGAT
GCTTGCACCGCGCGTGGCGTGTGGCTCACCTTCGTTCCAGATTTGCTGACCGTTCCAACCGCAGAACTGGCCATTGGTTTGGCT
GTTGGCTTGGGTCGCCACCTGCGTGCAGCCGATGCGTTCGTTCGCTCTGGCAAGTTCCGTGGTTGGCAGCCACGCTTCTACGGC
ACCGGTTTGGATAACGCAACCGTGGGCTTCTTGGGCATGGGTGCCATCGGTTTGGCTATGGCGGATCGCCTGCAGGGCTGGG
GTGCAACCCTGCAATACCACGAAGCAAAGGCCCTCGATACCCAGACCGAACAACGCCTGGGCCTCCGTCAAGTTGCATGCTCT
GAATTGTTCGCCTCCTCTGATTTCATTCTCTTGGCTTTGCCACTGAACGCGGATACCCTCCACTTGGTTAATGCTGAATTGCTCGC
ACTCGTTCGTCCAGGTGCTTTGCTGGTGAACCCATGTCGCGGTTCCGTGGTTGATGAAGCTGCGGTGTTGGCAGCCCTGGAAC
GTGGTCAGCTGGGCGGTTACGCTGCGGATGTTTTCGAAATGGAAGATTGGGCTCGCGCGGATCGTCCACAGCAAATCGATCCA
GCTCTCTTGGCGCACCCAAATACCCTCTTCACCCCACACATTGGCTCCGCAGTTCGCGCCGTGCGTTTGGAAATCGAACGCTGTG
CAGCCCAGAACATTCTCCAAGCATTGGCCGGTGAACGTCCAATCAACGCTGTGAATCGCCTCCCAAAGGCGAATCCAGCTGCG
GATTAA
